# Outbreak of Extended-Spectrum β-Lactamase–producing *Klebsiella oxytoca* Infections Associated with Contaminated Handwashing Sinks^1^

**DOI:** 10.3201/eid1808.111268

**Published:** 2012-08

**Authors:** Christopher Lowe, Barbara Willey, Anna O’Shaughnessy, Wayne Lee, Ming Lum, Karen Pike, Cindy Larocque, Helen Dedier, Lorraine Dales, Christine Moore, Allison McGeer, the Mount Sinai Hospital Infection Control Team

**Affiliations:** University of Toronto, Toronto, Ontario, Canada (C. Lowe, A. McGeer);; and Mount Sinai Hospital, Toronto (B. Willey, A. O’Shaughnessy, W. Lee, M. Lum, K. Pike, C. Larocque, H. Dedier, L. Dales, C. Moore, A. McGeer)

**Keywords:** Klebsiella oxytoca, outbreak, ICU, sink, extended-spectrum β-lactamase, ESBL, antimicrobial resistance, handwashing, bacteria

## Abstract

*Klebsiella oxytoca* is primarily a health care–associated pathogen acquired from environmental sources. During October 2006–March 2011, a total of 66 patients in a hospital in Toronto, Ontario, Canada, acquired class A extended-spectrum β-lactamase–producing *K. oxytoca* with 1 of 2 related pulsed-field gel electrophoresis patterns. New cases continued to occur despite reinforcement of infection control practices, prevalence screening, and contact precautions for colonized/infected patients. Cultures from handwashing sinks in the intensive care unit yielded *K. oxytoca* with identical pulsed-field gel electrophoresis patterns to cultures from the clinical cases. No infections occurred after implementation of sink cleaning 3×/day, sink drain modifications, and an antimicrobial stewardship program. In contrast, a cluster of 4 patients infected with *K. oxytoca* in a geographically distant medical ward without contaminated sinks was contained with implementation of active screening and contact precautions. Sinks should be considered potential reservoirs for clusters of infection caused by *K. oxytoca*.

## Abstract 2

Sinks are a potential reservoir for environment-to-patient and patient-to-patient transmission.

*Klebsiella oxytoca* is an opportunistic pathogen that causes primarily hospital-acquired infections, most often involving immunocompromised patients or those requiring intensive care. Reported outbreaks have most frequently involved environmental sources (*1**–**4*). *K. oxytoca*, like other *Enterobacteriaceae*, may acquire extended-spectrum β-lactamases (ESBL) and carbapenemases (*1**,**5*); outbreaks of multidrug-resistant *K. oxytoca* infection pose an increasing risk to hospitalized patients.

We report an outbreak of infections caused by ESBL-producing *K. oxytoca* in the intensive care unit (ICU), step-down unit, and medical care unit at a hospital in Toronto, Ontario, Canada, during a 4-year period. Contributing to the ongoing difficulties in the containment of this outbreak has been the contamination of handwashing sinks in the ICU. We describe a retrospective review of all *K. oxytoca* isolates intermediate or resistant to third-generation cephalosporins identified from inpatients from April 1997 through December 2011, the investigation of the source of the *K. oxytoca* outbreak, and the interventions implemented to contain the outbreak.

## Methods

The outbreak occurred at an acute tertiary-care facility in Toronto with 472 beds, including a 16 single-bed medical-surgical ICU, a 6-bed cardiac care unit , and two 4-bed step-down units. Outbreak cases of *K. oxytoca* were defined as hospital-acquired isolates with pulsed-field gel electrophoresis (PFGE) patterns belonging to 2 related clonal groups; all such isolates produced an Ambler class A ESBL. Isolates were considered hospital acquired if the first specimen (clinical culture or rectal swab) yielding resistant *K. oxytoca* was obtained >3 days after the admission date or if the specimen was obtained <3 days after admission in a patient who had been hospitalized at the outbreak hospital within the previous 3 months. Patients were characterized as infected or colonized on the basis of National Healthcare Safety Network definitions (*6*).

Hospital infection-control guidelines provide that each patient colonized or infected with class A ESBL-producing *Enterobacteriaceae* be moved to a separate, single room with contact precautions in place. Risk factor–based admission rectal swab screening for ESBL-producing *Enterobacteriaceae* was initiated in 2004. High-risk populations are all patients admitted to an ICU and general medicine or surgical patients being transferred from an acute or long-term care facility or having a history of recent hospitalization or colonization/infection with a multidrug-resistant organism. Periodic prevalence screening is undertaken on medical and surgical wards, and potential clusters of clinical ESBL-producing isolates are investigated. ESBL colonized or infected patients are flagged in an electronic clinical system. Additional precautions are continued until weekly rectal swab specimens are negative over 4 weeks, at which point patients are placed in private rooms with ongoing periodic screening for 6 months.

Clinical specimens were processed by using conventional microbiological techniques. The VITEK 2 system (bioMérieux, Marcy l’Etoile, France) was used for identification and antimicrobial drug susceptibility testing of *K. oxytoca* isolates. Rectal screening swabs were plated on MacConkey agar with cefpodoxime (2 μg/mL). Tap water was cultured by vigorously swabbing the inside of each faucet with a cotton swab, turning the tap on and collecting 50 mL of water, vortexing the tube containing the water and the swab, centrifuging the sample twice at 3,500 × *g* for 15 minutes, and resuspending the resulting pellet of precipitated material in 3 mL of brain–heart infusion broth. Other environmental samples were obtained by inoculating premoistened cotton swabs (for dry surfaces) or by adding ≈0.25 mL of gel/liquid to 3 mL of brain–heart infusion broth. For sink cultures, cotton swabs were used to sample ≈10 cm^2^ areas of the surface of the sink rim or basin. Drains were sampled by rotating swabs inserted 5–7 cm through the sink drain. Inoculated brain–heart infusion broth was incubated overnight at 37°C and then plated onto MacConkey agar with cefpodoxime. Clinical isolates intermediate or resistant to cefpodoxime (MIC >4 μg/mL) and colonies growing on the MacConkey agar with cefpodoxime underwent disk diffusion phenotypic confirmation (ceftriaxone, ceftazidime and aztreonam plus/minus clavulanic acid and cefoxitin) on Mueller-Hinton agar (*7*). PFGE was performed by using the restriction enzyme *Xba*I, with a run time of 20 h and switch times of 5 to 35 s at 12°C and 6 V/cm (CHEF-DR II System; Bio-Rad, Hercules, CA, USA); profiles were analyzed by using BioNumerics (Applied Maths, Sint-Martens-Latem, Belgium).

## Results

### Outbreak Description

Isolation of ESBL-producing *K. oxytoca* was uncommon in the 9 years before the outbreak; from January 1, 1997, through September 30, 2006, 10 clinical isolates (no bacteremias) and 6 colonized patients were identified. All but 1 colonized patient acquired the organism in the hospital, and 16/19 (84.2%) patients were previously or currently admitted to the ICU at the time of culture. PFGE of these isolates revealed that 5 (26.3%) isolates belonged to pattern A, 3 (15.8%) isolates belonged to pattern B, 3 isolates were closely related to each other but unrelated to isolates of pattern A or B, and 3 isolates had unique patterns. Two isolates were unavailable for typing. Only 1 case (April 2004) was identified between April 2003 and September 2006.

**Figure 1:**
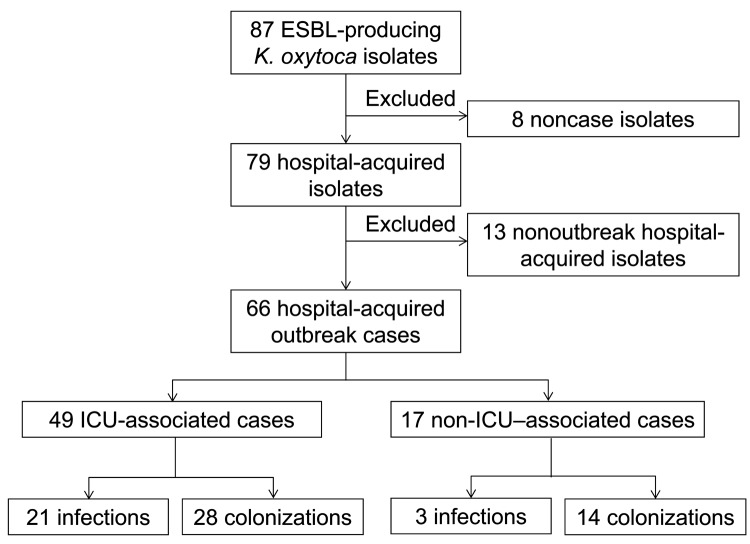
Flow of extended-spectrum β -lactamase (ESBL)–producing *Klebsiella oxytoca* infection and colonization in patients at a hospital in Toronto, Ontario, Canada, October 2006–March 2011. ICU, intensive care unit.

From October 2006 through March 2011, ESBL-producing *K. oxytoca* was isolated from 87 patients (Figure 1); 21 were not part of the outbreak. Eight of these nonoutbreak patients had isolates from clinical cultures or screening specimens obtained within 72 hours of first admission to the hospital, but each isolate had a unique pattern by PFGE. The remaining 13 had isolates first identified >3 days after admission (n = 11) or had been previously admitted to this hospital (n = 2), and each isolate had a unique PFGE pattern, with no temporal or geographic clustering. The remaining 66 patients were classified as outbreak case-patients.

All 66 outbreak case-patient isolates carried Ambler class A β-lactamases. Clinical *K. oxytoca* isolates were identified from 27 patients; among these, 24 patients had 25 hospital-acquired infections (9 urinary tract infections, 4 of them bacteremic; 8 asymptomatic bacteriurias; 4 soft tissue infections, 1 of them bacteremic; 3 primary bacteremias; and 1 pneumonia with bacteremia). Of the 9 bacteremias, 8 were PFGE pattern A. On the basis of study definitions, 3 patients had clinical specimens (2 sputum samples and 1 bronchoalveolar lavage culture) that were not associated with infection. In 11 cases, clinical cultures were preceded by identified rectal colonization; median time to first identification of a clinical isolate after recognition of colonization was 10 days (mean 12.5 days, range 1–31 days). Of the remaining 16 cases with clinical isolates of *K. oxytoca*, 13 patients had a prior negative result from an ESBL rectal screening. Thirty-nine patients were identified as colonized by rectal swab screening but no subsequent clinical isolate was identified. Patients remained colonized for variable periods of time, with the proportion colonized still positive on repeat screening as follows: 7 days (30/49, 61.2%), 14 days (20/41, 48.7%), 21 days (15/38, 39.4%), 28 days (14/33, 42.4%), 2 months (6/22, 27.3%), and 3 months (4/17, 23.5%).

**Figure 2:**
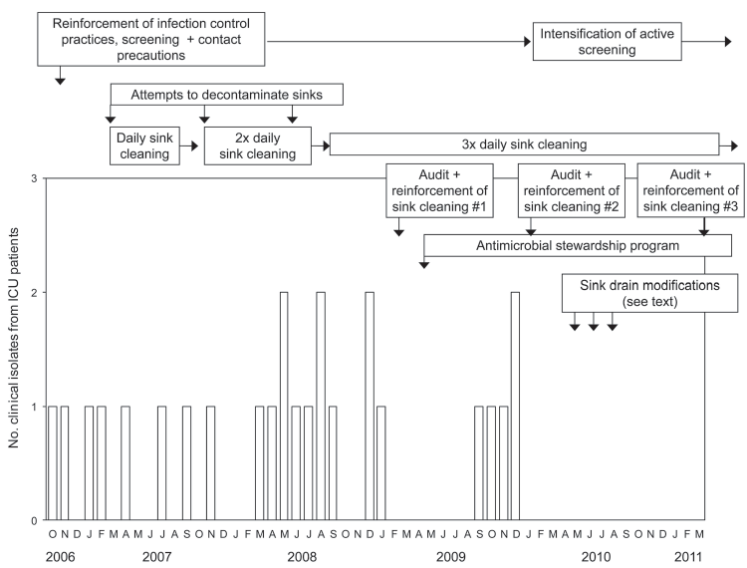
Nosocomial extended-spectrum β-lactamase–producing *Klebsiella oxytoca* clinical isolates from patients in the intensive care unit of a hospital in Toronto, Ontario, Canada, and the associated interventions implemented to contain the spread of the outbreak, October 2006–March 2011. ICU, intensive care unit.

Figure 2 summarizes the occurrence of outbreak-related ESBL-producing *K. oxytoca* clinical isolates over time in the ICU, where most cases occurred (49/66, 74%). Cases with clinical isolates were identified regularly from October 2006 through December 2009; however, no clinical isolates have been recovered from patients in or exposed to the ICU since that time. The 6 outbreak cases in 2010 and 4 in 2011 were identified only by rectal swab screening. The number of newly identified cases on point-prevalence screens was 13/1,049 in 2008 (1.2%), 7/1,744 (0.4%) in 2009, 6/921 (0.7%) in 2010, and 1/754 (0.1%) in 2011 (p = 0.01). Because prevalence screens were performed more frequently when new cases were occurring, the total number of prevalence screens decreased in 2010 and 2011 compared with 2008 and 2009.

Seventeen case-patients who had not been admitted to the ICU were identified as colonized or infected with outbreak strains. Eleven of these were part of 3 clusters. In August 2010, a total of 4 patients (3 colonized, 1 infected) from whom isolates were identified that were indistinguishable by PFGE (pattern A) acquired the strain on a single medical ward. In the medical step-down unit, 3 patients were identified as becoming colonized during June–September 2010; an additional 4 patients became colonized in February and March of 2011 (all pattern A). The remaining cases were sporadically identified on the surgical step-down unit (n = 4) and in general surgery/gastroenterology units (n = 2).

### Outbreak-specific Infection-control Interventions

At the onset of the outbreak (October 2006), the investigation encompassed a search for potential environmental sources. Samples for culture were obtained from potential reservoirs (e.g., shared equipment such as electrocardiogram and ultrasound machines, bronchoscopes and solutions used in endoscopy areas, glucometers, hand creams, lubricating gels, disinfectant swabs, blood gas machines, water baths, ice machines, mouthwashes, oral medications, and soaps), and prevalence screening was conducted to detect patient colonization in the ICU. No environmental sources were identified, no multidose vials or bags of parenteral fluids or oral medications were in use in the unit, and no procedures or exposures were identified that linked affected patients but not other patients. Two of 16 sinks had aerators, neither of which yielded *K. oxytoca* on culture. Tap water and sinks were not cultured at this time. All patients newly identified as colonized or infected had contact precautions applied. Hand hygiene practices, adherence to contact precautions, and appropriate management of water/liquid/gels were reinforced. These interventions had no apparent effect on the rate of new clinical infections; new patients acquired the organism after weeks without identified colonized patients in the ICU.

In April 2007, results of repeat screening of potential environmental sources were negative, as were samples of tap water; however, multiple handwashing sinks were found to be contaminated with the outbreak strains. Sinks in the ICU are foot pedal–operated, free-standing, porcelain hand hygiene sinks located just inside the door to each ICU room. Although intended only for hand hygiene, they were also used for disposal of fluids, including body fluids. When sinks were identified as a potential reservoir, use of the sinks for hand hygiene only was reinforced. Attempts were made to reduce or eradicate *K. oxytoca* contamination by cleaning sinks and leaving them unused for 48 hours with disinfectant standing in traps. When this process failed, routine daily sink disinfection was initiated; sink surfaces, including taps, rims of sinks, and basins, were cleaned with a 1:16 dilution of Virox (Virox Technologies Inc., Oakville, CA, USA), and ≈250 mL of the diluted solution was poured down the drain. Neither this daily cleaning, nor month-long trials of cleaning with bleach and with a foaming hydrogen peroxide product, resulted in reduced sink colonization rates. Sink cleaning was increased to 2×/day in late 2007 and 3×/day in August 2008. Adherence to cleaning standards, particularly frequency of cleaning, was variable. Regular reminders to cleaning staff were required, and the identification of new hospital-acquired cases usually resulted in recognition that adherence had decreased.

**Figure 3:**
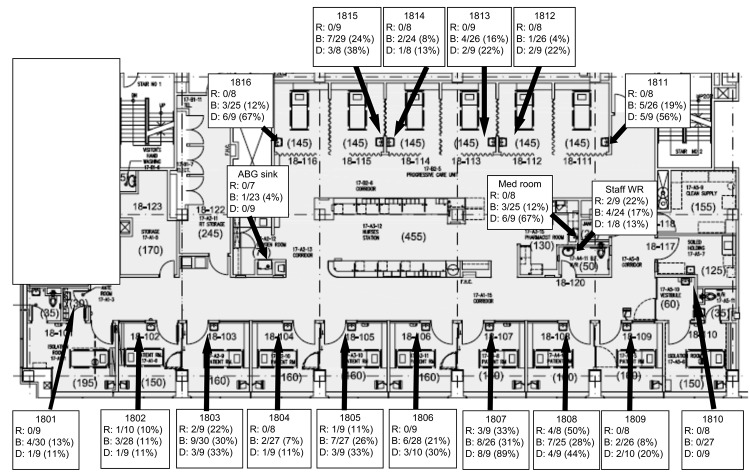
Locations of environmental screening for extended-spectrum β-lactamase–producing *Klebsiella oxytoca* in the intensive care unit sinks. Numbers indicate room numbers. R, sink rim; B, sink basin; D, sink drain; WR, washroom; med room, medication room (pharmacy); ABG, arterial blood gas room.

Figure 3 shows the overall rates of recovery for patients with outbreak-related ESBL-producing *K. oxytoca* infection associated with handwashing sinks in the ICU. ICU sink culture screens were performed on 29 separate occasions, yielding a total of 910 cultures. The average rate of sink contamination during the outbreak period was 16.4% (149/910). After implementation of 3×/day cleaning/disinfection of sinks (October–December 2008), the sink colonization rate decreased to 3.9% (3/77) during the quarter; the rate increased to 16.7% (71/424) the following quarter (January–March, 2009), when adherence to routine sink cleaning was noted to have decreased.

Many of the ICU sinks had old patented opening drains (a pipe connecting the sink basin to the sink trap), a design that allowed drainage from the overflow hole to mix with the regular drainage water, potentially impairing adequate drainage. During February–June 2010, all drains were changed, eliminating the connection with the overflow drain; the overflow holes were decommissioned; the strainers in the sink basin were replaced by strainers containing a larger number of smaller holes to reduce backsplash; and sink traps were replaced.

Investigation of the medical unit on which 4 patients acquired outbreak strains during summer 2010 failed to identify an environmental source; all sink cultures were negative. After initiation of standard contact precautions for the colonized patients, no additional colonized or infected patients were identified in that unit. In contrast, the outbreak strain of ESBL-producing *K. oxytoca* was recovered from sinks, but not other environmental sources, in the medical step-down unit during August 2010. The implementation of regular sink cleaning and contact precautions for colonized patients resulted in no cases being identified during September 2010–February 2011. When new cases were identified in 2011, the previously described sink modifications were implemented in the step-down unit.

### Concomitant Infection-control Interventions

As part of an ongoing program to improve adherence to hand hygiene, routine observational hand hygiene audits started throughout the hospital in fall 2008. Compliance rates improved gradually over the course of the outbreak, from 59.4% in 2008 to 69.8% in 2011.

An antimicrobial stewardship program was initiated in only the ICU in February 2009. An audit and feedback program was instituted and run by an infectious diseases physician and a pharmacist. During the first year of the program, the mean antibacterial defined daily dose per 100 patient-days decreased 9.2% compared with the same time period in the previous year (*8*).

## Discussion

Outbreaks of health care–associated infection caused by *K. oxytoca* have most often been associated with contamination of environmental reservoirs such as disinfectants (*9*), multidose vials or parenteral fluid bags (*10**,**11*), humidifiers (*2*), and ventilators (*3*). This outbreak suggests that handwashing sinks in high-intensity hospital care areas may be a reservoir for *K. oxytoca* and that person-to-person transmission may also occur.

Patients in this medical-surgical ICU continued to acquire the outbreak organism despite review of routine practices, hand hygiene education and auditing, screening to identify colonized patients, and implementation of contact precautions for colonized and infected patients. In contrast, this approach seemed to control transmission in the medical ward, where contamination in sinks was not found, and has been reported to be successful in the control of other outbreaks of ESBL-producing *Enterobacteriaceae* (*12*). In this hospital, transmission of the outbreak strains of *K. oxytoca* seemed to occur both from sinks and from colonized/infected patients. As the emergence of carbapenemase-producing organisms focuses attention on health care–associated infections due to *Enterobacteriaceae,* other reservoirs may also be recognized. Recently, a clone of *K. pneumoniae* possessing SHV-1 and CTX-M-15 ESBLs was implicated in a hospital-wide foodborne outbreak in Spain, in which the hospital kitchen and colonized food handlers were the presumed reservoirs (*13*).

During the outbreak reported here, patients who acquired ESBL-producing *K. oxytoca* colonization were followed up with routine rectal swab screening; 23.5% remained colonized after 3 months. Colonization with ESBL-producing *Enterobacteriaceae* can persist for months to years (*14**,**15*); data are insufficient to determine whether duration of carriage is different for different species or clones of *Enterobacteriaceae*. In 1 study, only 6.8% of colonized patients cleared carriage over 3 years of follow-up (*14*). In addition, colonized patients may have intermittently positive rectal screening results, which suggests carriage at concentrations below the limit of detection for rectal swab specimens (*16*).

At the hospital in this study, patients to whom contact precautions are applied remain under these guidelines for 1 month and in private rooms for 6 months. However, for resistant gram-negative bacteria of epidemiologic importance (e.g., carbapenemase-producing organisms), extending the duration of contact precautions until discharge may be warranted. Because of the long duration of colonization, hospitalization is also likely to be a risk factor for community-onset infection with multidrug-resistant *K. oxytoca*, as has recently been described in Athens, Greece (*17*). The existence of asymptomatic colonized patients compounds the difficulty of containing the spread of these organisms; containing outbreaks without active surveillance may not be possible (*18*).

The outbreak-associated clones of *K. oxytoca* found in this study were ubiquitous in sinks in the ICU, cultured from 15/16 patient rooms as well as from other sinks (e.g., staff washrooms). Increased sink cleaning and auditing was associated with a decline in clinical isolates, but these measures proved difficult to sustain. Achieving persistent reductions in the degree of contamination in ICU sinks is difficult but has been a necessary intervention in outbreaks of *Pseudomonas aeruginosa* (*19**,**20*). In these outbreaks, structural changes, including renovation to sinks and plumbing or alteration of water temperature, reduced but did not eliminate the outbreak organism from sink drains. As in our experience, although the organisms could still be recovered after alterations to improve drainage and reduce splashing, these modifications were temporally associated with persistent declines in the rate of clinical infections. Persistence of *Pseudomonas* spp. in ICU sinks has been attributed to biofilm formation, which allows stable attachment to environmental surfaces and protection from disinfection (*20**,**21*). Biofilm formation has also been described for *K. oxytoca* on filtration membranes (*22*) and is probably a factor in the persistence of *K. oxytoca* in sinks in this outbreak.

This outbreak also emphasizes the challenges associated with limited space and sinks in older hospitals. Presumptively, these handwashing sinks became contaminated because they were used for the disposal of body fluids from colonized patients. While this is clearly unacceptable, nurses in the ICU are required to walk past several rooms (and out of isolation rooms) to reach the dirty utility room for disposal of body fluids, an activity that is also associated with risk. As we increasingly recognize the risks associated with hospital water and sinks, the design of ICUs becomes critical for protecting patients from these risks.

In conclusion, we describe an outbreak in which colonized sinks were a contributing reservoir for ESBL-producing class A *K. oxytoca.* A multifaceted approach including reinforcement of infection control policies (hand hygiene, contact precautions, isolation and admission/routine rectal screening, clear delineation between handwashing sinks and sinks for other purposes), intensified cleaning of sinks, structural changes to the sinks, and antimicrobial stewardship has reduced but not eliminated transmission of the outbreak strain. Although *K. oxytoca* is in the family *Enterobacteriaceae*, its epidemiology is not clearly defined, and it may be more likely than other *Enterobacteriaceae* to be associated with environmental reservoirs in hospitals. Sinks should be considered potential reservoirs when clusters of infection caused by *K. oxytoca* are investigated.
